# Comparative Evaluation of Stress Distribution and Deformation in Class II Cavities Restored With Two Different Biomimetic Restorative Materials: A Three-Dimensional Finite Element Analysis

**DOI:** 10.7759/cureus.69179

**Published:** 2024-09-11

**Authors:** Parul Bansal, Tanya Seth, Mohit Kumar, Megna Bhatt, Pulkit Arora, Iti Gupta, Swati Chaudhary, Sravyanjali Akkanapally, Ankusha Arora, Shefali Singh

**Affiliations:** 1 Department of Conservative Dentistry and Endodontics, Shree Bankey Bihari Dental College and Research Centre, Ghaziabad, IND; 2 Department of Dentistry, Kalyan Singh Government Medical College, Bulandshahr, IND

**Keywords:** activa bioactive, biomimetic restorative material, everx posterior, finite element analysis, stress distribution, total deformation, von-mises stress

## Abstract

Aim

This study aims to comparatively evaluate the stress distribution and deformation in class II cavities restored with EverX posterior and Activa^TM^ Bioactive restorative material using three-dimensional finite element analysis.

Methods

On a mandibular first molar tooth model, features of a class II mesio-occlusal cavity were designed. Following the application of EverX Posterior and Activa^TM^ Bioactive, the model was subjected to a 600 N static occlusal load. Three-dimensional finite element analysis was used to analyze the distribution of occlusal stresses among the remaining components and restorative materials. von Mises and maximum principal stresses were evaluated and compared.

Results

von Mises and maximum principal stresses of Activa^TM^ Bioactive components were higher compared to the control group and EverX Posterior model. Activa^TM^ Bioactive restorations had absorbed more stresses within the material rather than distributing it to enamel and dentin than EverX Posterior material. Also, deformation was extremely high in the Activa^TM^ Bioactive model, which may be because of less stiffness of the material.

Conclusion

EverX posterior has a higher capacity to bear stresses in stress-bearing areas than Activa^TM^ Bioactive Restorative.

## Introduction

Following dental treatment, the healthy teeth, the supporting bone, the soft tissues, and the teeth that have been adhesively healed may all experience stress from functional and parafunctional pressures in the tooth. Research has consistently shown that identifying the distribution and analyzing these stresses can significantly lower the chance of dental restoration failure [1,2]. Class II restorations must be able to withstand the forces exerted on them [1].

These days, stress may be mitigated by the application of several biomimetic restorative materials, either alone or in combination. Biomimetic restorative materials are materials used in dentistry to restore tooth structure and function by mimicking natural tissues. A new material called EverX Posterior is made up of barium glass filler and short E-glass fibers, with the pre-incorporated glass fiber length being 1-2 mm. These short fibers aid in halting the crack progression in a way similar to dentine’s function [3].

By replicating natural teeth’s physical and chemical properties, Activa^TM^ Bioactive-restorative material combines the benefits of glass ionomers with composites’ durability and esthetics [4]. The three main ingredients are bioactive ionomer glass, patented rubberized resin, and patented bioactive ionic resin. Bioactive ionomer glass has a high fluoride release near the tooth restoration interface. As a result, it can manifest in a broad range of ways, from traditional class I, class II, and class V caries to intricate carious lesions encompassing several surfaces [5].

Three-dimensional finite element analysis is a mathematical technique that builds models in a virtual environment and applies different kinds of intricate mechanical stresses to them [6]. It aids in the analysis of the stress pattern at any tooth position that is challenging to document clinically [6]. Reliable analysis requires three-dimensional modeling of the teeth due to their non-symmetrical form, alveolar bone, periodontal ligament, and many other associated components [1]. Based on the internal stresses of a system, as shown by the finite element analysis, predictions regarding fracture susceptibility can be generated [7].

We employed three-dimensional finite element stress analysis to compare biomimetic restorative materials’ stress distribution under simulated occlusal loading. Biomimetic restorative materials were selected for the study, as these materials have properties similar to those of dentin and can be used for restoration in stress-bearing areas and to overcome clinical complications. The mandibular first molar has a dentin-embedded class II mesio-occlusal cavity, proximal box, and gingival floor.

## Materials and methods

Model preparation for finite element analysis

The study aimed to understand the behavior of the tooth under loads for the controlled model (intact model), restored tooth with the EverX posterior material, and similarly, an additional model restored with Activa^TM^ Bioactive.

Since the FEA requires a very clean and error-free model to perform the simulations with no uncertainties in the results achieved, extra care was taken from the beginning in terms of taking the average size of the tooth with no caries and no surface defects. The process started with taking an extracted human mandibular first molar tooth, recently extracted for periodontal issues, which was scanned with CBCT using Morita 3D Accuitomo 170 (J Morita Mfg. Corp., Kyoto, Japan). This scan resulted in a DICOM file. In this stage, it was required to convert the DICOM file into a mesh file (STL format). The conversion of the process started with performing segmentation using the thresholding approach, as the difference in properties of the enamel, dentine, and pulp results in different intensities. This intensity was used as a reference for segmentation, and the components of the tooth were extracted into STL format.

Extracted STL is known to have many surface issues as the Mimics program (Mimics 12.00, Leuven, Belgium) is not equipped with surface or solid body treatment options to resolve arising mesh issues. A mesh mixer was utilized to fix the sharp corners. After cleaning the files in the mesh mixer, the model was then imported into Recap photo and Fusion 360 to convert into the solid body, which had the sequence of the process of mesh conversion involving tri mesh to quad mesh (obj) conversion in Recap photo and followed by T-spline to the solid body with the help of Fusion 360. The model was then processed using the SolidWorks software to produce restorations. Finally, the models were imported into ANSYS Workbench (Ansys, Inc., Canonsburg, PA, USA) software, and an FEA study was made to compare and analyze the behavior of the restorative materials when placed inside the tooth cavity.

Each model structure has its own set of established physical attributes, including elasticity modulus and Poisson ratio. The tooth’s final scaling and modeling were completed. The model that served as the control group was the one with the intact lower first molar teeth [1]. In the model, the periodontal ligament had a thickness of 0.2 mm, and the cortical bone had a thickness of 2 mm [1].

Class II mesio-occlusal cavity was prepared on the permanent first molar in SolidWorks by using the Boolean operations. The cavity was prepared with certain dimensions, such as a gingival floor of 1 mm below the cemento-enamel junction, a buccolingual width measuring about 2.6 mm, and a gingival width of the proximal box of 1.5 mm [1]. The dimensions of the two restored models remain the same except for the materials, which are changed in the FEA simulation software (Ansys, Inc.).

It was presumed that all materials and oral tissues utilized would be linear and homogeneous. All materials were considered to be isotropic in this study as it was assumed that the physical behavior and property remained the same in all directions. The element type of the FEA model was tetrahedron having 10 nodes. The mechanical properties of dental tissues and restorative material used in the study are described in Table 1. 

**Table 1:** Mechanical properties of dental tissues and restorative materials

Properties\material	EverX Posterior	Activa^TM^ Bioactive	Enamel	Dentin	Pulp
Elastic modulus (in GPa)	11.4	2.35	84.1	18.6	2.0
Poisson ratio	0.24	0.25	0.33	0.32	0.45

Grouping of models

Three models were considered as follows:

Group 1: Virtual controlled model

Group 2: Virtual model restored with EverX Posterior

Group 3: Virtual model restored with Activa^TM^ Bioactive

Load application

A 600N force is considered the average maximum masticatory load applied on the tooth; hence, it was delivered to the virtual models’ occlusal area at a 60° angle to their long axis, as per Alp et al.’s 2020 study [1]. A linear static type of analysis was performed for which boundary conditions were set, and von Mises and principal maximum stresses were assessed independently for each model on residual dentin, restorative materials, and buccal and lingual enamel. As it is an observational finite element analysis study, hence no statistical methods were applied. The measured values were evaluated and compared.

## Results

**Figure 1:**
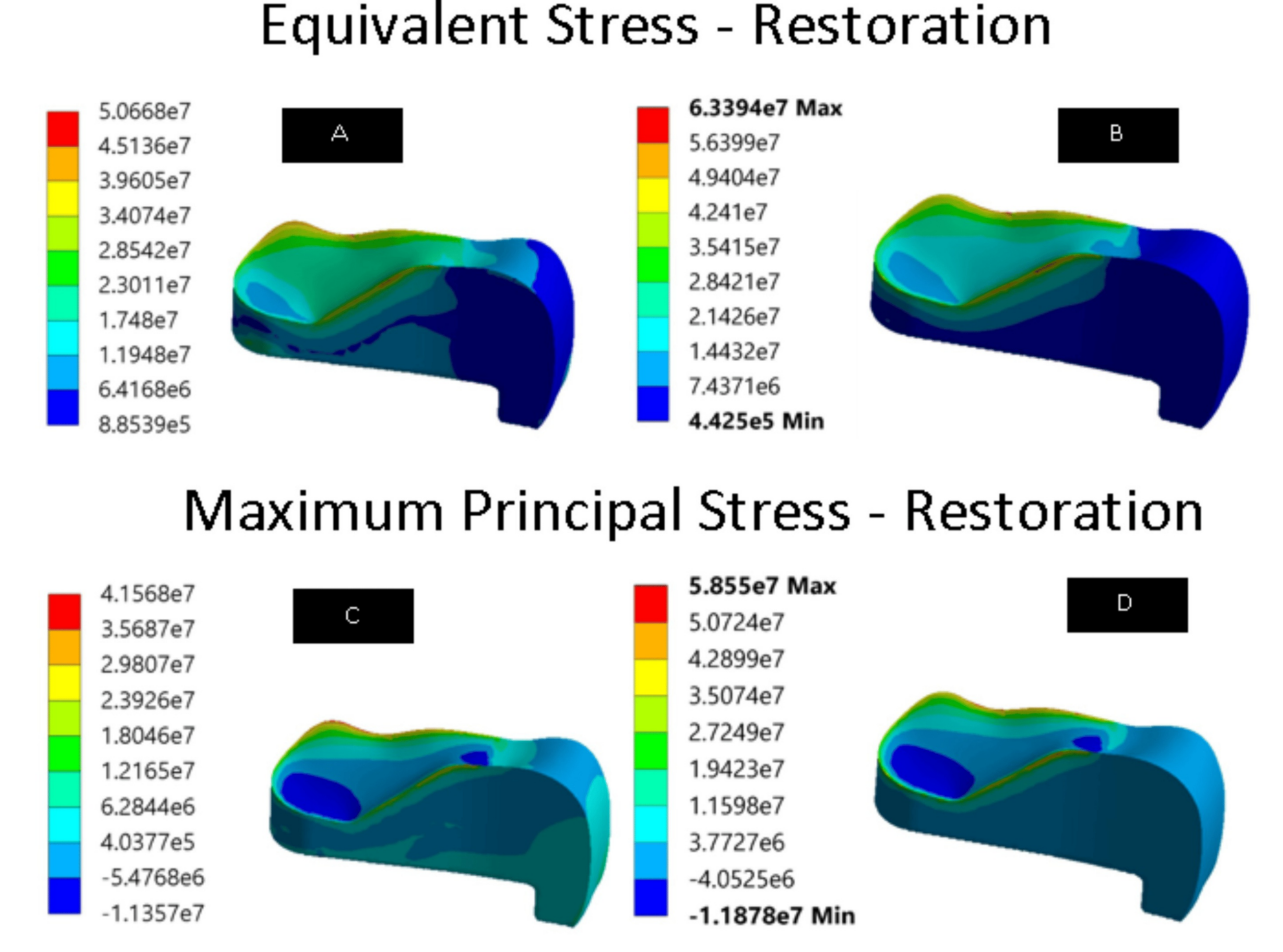
Equivalent and maximum principal stresses of the restoration (unit: pascals) (A) Equivalent stress of EverX Posterior restorative material. (B) Equivalent stress of Activa^TM^ Bioactive restorative material. (C) Maximum principal stress of EverX Posterior restorative material. (D) Maximum principal stress of Activa^TM^ Bioactive restorative material.

**Figure 2:**
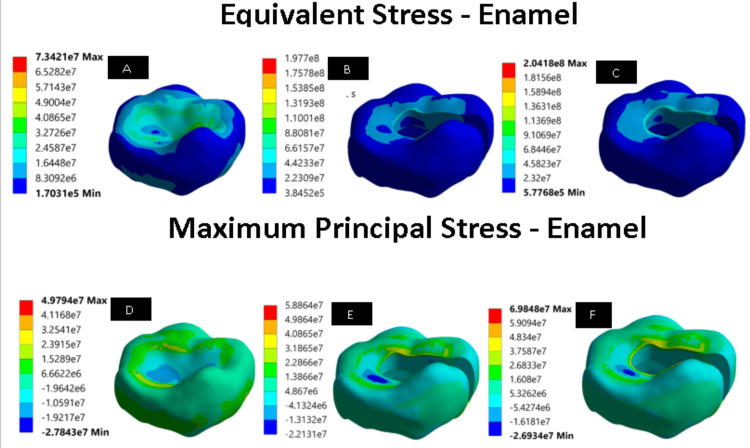
Equivalent stress and maximum principal stress of all the models with enamel (unit: pascals) (A) Equivalent stress of controlled model (intact model) with enamel. (B) Equivalent stress of EverX Posterior restorative material with enamel. (C) Equivalent stress of Activa^TM^ Bioactive restorative material with enamel. (D) Maximum principal stress of controlled model (intact model) with enamel. (E) Maximum principal stress of EverX Posterior restorative material with enamel. (F) Maximum principal stress of Activa^TM^ Bioactive restorative material with enamel.

**Figure 3:**
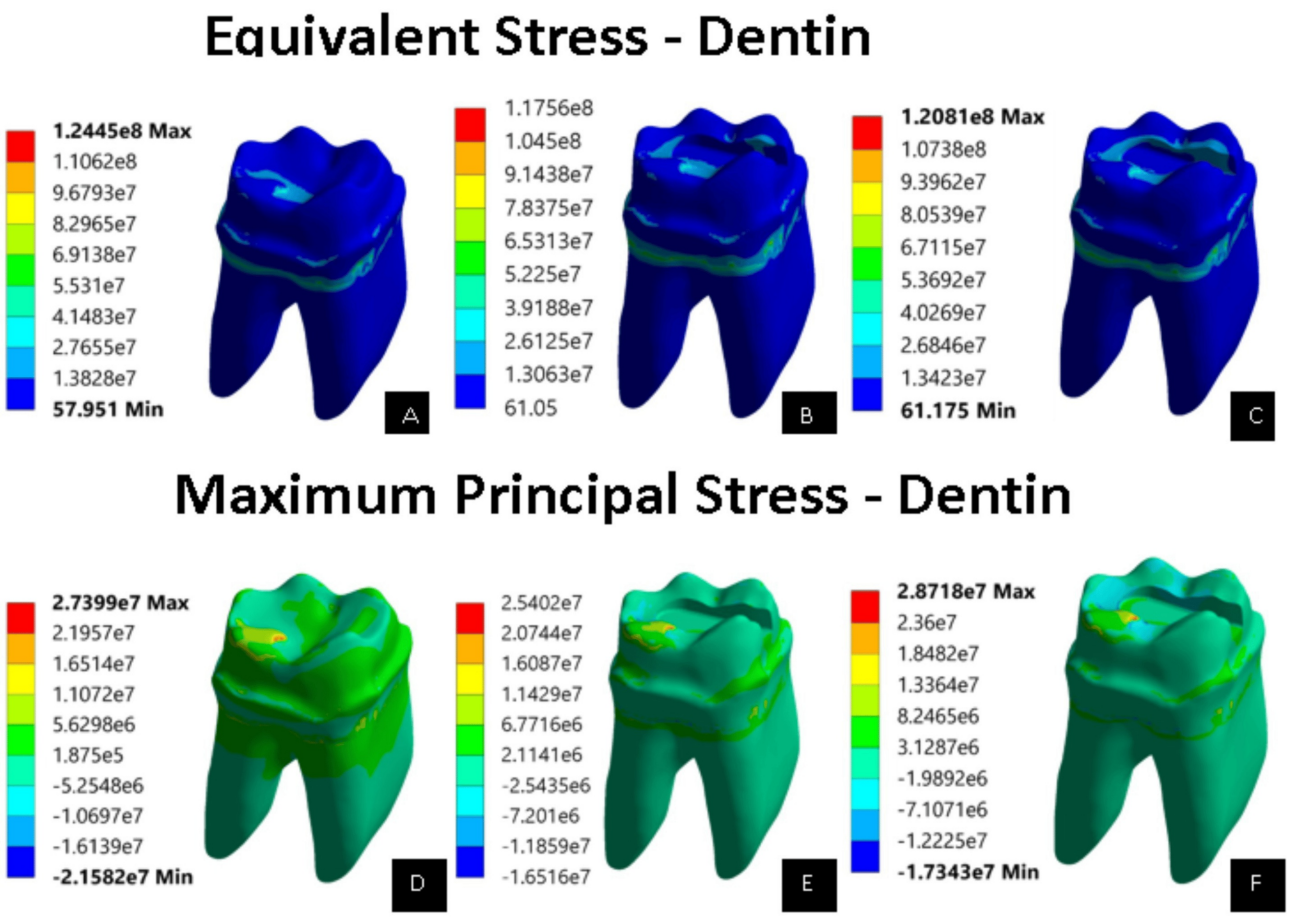
Equivalent stress and maximum principal stress of all the models with dentin (unit: pascals) (A) Equivalent stress of controlled model (intact model) with dentin. (B) Equivalent stress of EverX Posterior restorative material with dentin. (C) Equivalent stress of Activa^TM^ Bioactive restorative material with dentin. (D) Maximum principal stress of controlled model (intact model) with dentin. (E) Maximum principal stress of EverX Posterior restorative material with dentin. (F) Maximum principal stress of Activa^TM^ Bioactive restorative material with dentin.

We ran the simulations using the specified loading conditions. The main aim of the research was to evaluate the principal and von Mises stress of the restoration. The present study focused on how well the two materials worked separately. The overall consolidation of the results is shown in Table 2. Group 3 had a greater stress value than group 2, as measured by the maximum principal and the von Mises stresses (Figure 1). The values for the principal and von Mises stress of the enamel indicated that group 1 had lower stresses (Figure 2). The stress values in group 2 and group 3 were different, with group 3 having a higher stress value of 204.1 MPa, while group 2 measured about 197.7 MPa. While comparing the stress values of dentin, group 1 had higher stresses than group 2 and group 3 (Figure 3). Group 3 had a higher value (120.8 MPa) than group 2 (117.5 MPa). From the pattern, it can be observed that the dentin component faces a high amount of stress on the line where it interacts with the restoration. Also, the stress in the contact region was higher.

**Table 2:** Overall deformation, principal stress, and von Mises stress values of all models

S. no	Component	Controlled model				EverX Posterior				Activa^TM^ Bioactive			
		Total deformation (in m)	von Mises stress (in Pa)	Max. principle stress (in Pa)	Min. principle stress (in Pa)	Total deformation (in m)	von Mises stress (in Pa)	Max. principle stress (in Pa)	Min. principle stress (in Pa)	Total deformation (in m)	von Mises stress (in Pa)	Max. principle stress (in Pa)	Min. principle stress (in Pa)
1	Tooth	5.105e-6	1.244e8	4.979e7	-2.784e7	7.151e-6	1.977e8	5.886e7	-2.213e7	1.617e-5	2.041e8	6.984e7	-2.693e7
2	Enamel	-	7.342e7	4.979e7	-2.784e7	-	1.977e8	5.886e7	-2.213e7	-	2.041e8	6.984e7	-2.693e7
3	Dentin	-	1.244e8	2.739e7	-2.158e7	-	1.175e8	2.540e7	-1.651e6	-	1.208e8	2.871e7	-1.734e7
4	Restoration	-	-	-	-	-	5.066e7	4.156e7	-1.136e7	-	6.339e7	5.855e7	-1.187e7

**Figure 4:**
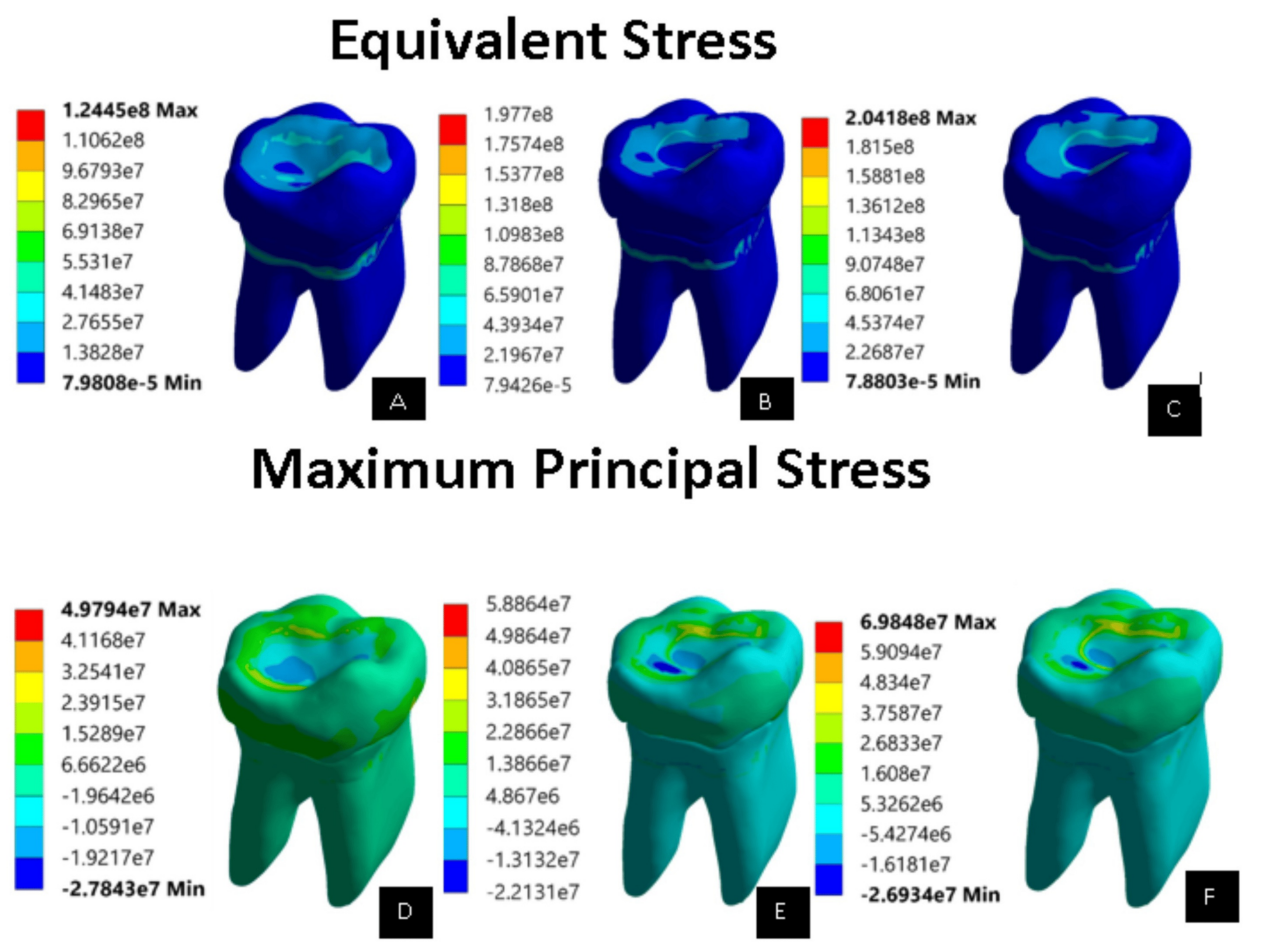
Equivalent stress and maximum principal stress of all the complete models (unit: pascals) (A) Equivalent stress of complete controlled model (intact model). (B) Equivalent stress of EverX Posterior restorative material on complete model. (C) Equivalent stress of Activa^TM^ Bioactive restorative material on complete model. (D) Maximum principal stress of complete controlled model (intact model). (E) Maximum principal stress of EverX Posterior restorative material on complete model. (F) Maximum principal stress of Activa^TM^ Bioactive restorative material on complete model.

**Figure 5:**
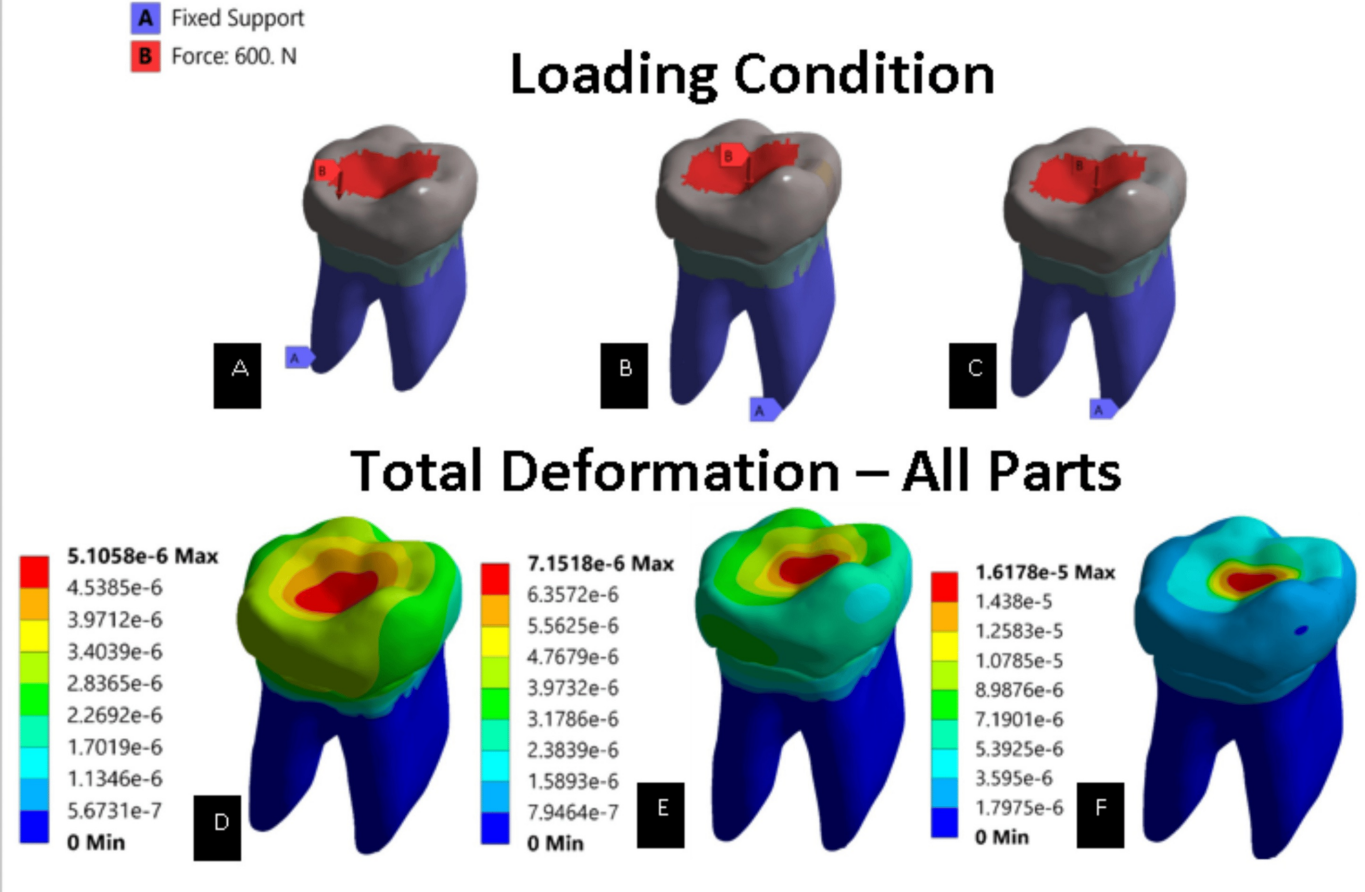
Load conditions and total deformation obtained in all parts (unit: meter) (A) Load conditions of the controlled model (intact model). (B) Load conditions of model restored with EverX Posterior restorative material. (C) Load conditions of model restored with Activa^TM^ Bioactive restorative material. (D) Total deformation of complete controlled model (intact model). (E) Total deformation of model restored with EverX Posterior restorative material. (F) Total deformation of model restored with Activa^TM^ Bioactive restorative material.

On comparing von Mises stress and principal stress of the complete virtual models (Figure 4), group 3 values (2.041e8) were higher than group 1 (1.244e8) and group 2 (1.977e8). The total deformation results obtained showed that group 1 had a maximum of 5.105e-6 m deformation, which in scale is as low as 0.00510 mm (Figure 5). Similarly, results for group 2 and group 3 were taken and observed to have 0.00715 and 0.01617 mm, respectively. These results showed on the comparison that group 1 had the least deformation, while group 3 had extreme deformation.

## Discussion

Fractures are common in class II cavities because of the significant amount of tooth tissue that is lost, primarily as a result of marginal ridge loss. Reduction in tooth strength can reach 46% when one marginal ridge is removed and 63% when two ridges are removed [8]. A large body of evidence suggests that stresses acting perpendicular to the tooth’s axis are less damaging than oblique stresses [9]. Fatigue life and the material’s performance are both affected by poorly distributed stresses over extended periods of time [8].

Assessment of stress distribution to the different portions of the tooth and their behavior is necessary to examine various biomechanical aspects, such as modulus of elasticity, Poisson’s ratio, etc., of the tooth and restoration, leading to more predictable clinical outcomes [10]. FEA is a numerical method for assessing and examining patterns of stress distribution [11]. Hence, it was used in the study as it helps to visualize how stress is distributed over different restorations.

The comparison of von Mises and principal stresses of the restoration was the major key of the study, which showed that the stress values in teeth restored with EverX Posterior and Activa^TM^ Bioactive were different, with Activa^TM^ Bioactive having higher stress values. From the pattern, it can be observed that the restoration component faces a high amount of stress on the line where it interacts with the tooth tissues. This shows that a change in the modulus of the material used may have a difference in the stress distribution. EverX Posterior may have performed better because of the uniform stress distribution caused by its identical modulus of elasticity to the surrounding dentin [12]. Composite restorations’ suitability for use with high or low elasticity moduli is debatable. The study conducted by Ausiello et al. found that using restorative and low-modulus luting materials can help minimize stress intensity by partially absorbing deformations during loading [13]. Another study by He et al. stated that a higher modulus of elasticity leads to higher stress concentration in the restoration [14].

The von Mises and primary stresses of enamel and dentin showed how restorative materials interacted with them. Activa^TM^ Bioactive repaired teeth showed greater stress values than EverX Posterior with enamel and dentin. From the pattern, it can be observed that the enamel and dentin component faces a high amount of stress on the line where it interacts with the restorative materials.

The deformation of a structure or material plays a crucial role in deciding the choice of restorative material in stress-bearing areas of the tooth. Analyzing the total deformation and the values for von Mises and principal stresses on complete virtual models showed that the controlled model had the least deformation and stress values, while teeth restored with Activa^TM^ Bioactive had extreme deformation and higher stress values than teeth restored with EverX Posterior. This may be due to the less stiffer behavior of Activa^TM^ Bioactive than EverX Posterior. The EverX posterior’s short e-glass fibers stop and halt the spread of cracks, which frequently begin near the restoration’s surface [15]. According to a previous study, the stress distribution pattern in the remaining tooth structure can change depending on the thickness of the restorative material used [16]. Research conducted by Abdulamir and Majeed [17] showed that teeth might be made more resistant to fractures when direct composite restorations are reinforced with e-glass fiber.

The longevity and retention of cavities is a day-to-day challenge for dentists. For this reason, it is essential that dental restorations should have mechanical qualities that can endure the strains and stresses produced during repetitive mastication.

The study has certain limitations. Restorative materials used in this study showed ideal behavior in the restored models, whereas in the actual oral environment, it differs by various factors such as polymerization shrinkage, cavity margins, setting time, etc. Since the FEA study is based on the analysis of the virtual model’s behavior and its results may differ from the actual clinical conditions, further research is needed to observe the behavior of these above-mentioned restorative materials in the oral environment for better success of the restorative treatment.

## Conclusions

The study concludes that within the constraints of this 3D FEA, it was determined that Activa^TM^ Bioactive restorations had absorbed more stresses within the material rather than distributing it to enamel and dentin as compared to EverX Posterior material. Also, deformation was extremely high in the Activa^TM^ Bioactive model, which may be because of less stiffness of the material. Therefore, in clinical scenarios, EverX Posterior can be used as a choice of material in stress-bearing areas because it enhances clinical performance through its even stress distribution and longevity of restoration.
